# Dosimetric Predictors of Acute Radiation Pneumonitis and Esophagitis in Hypofractionated Thoracic Irradiation of Non-Small Cell Lung Cancer Patients With Poor Prognostic Factors

**DOI:** 10.1016/j.adro.2024.101682

**Published:** 2024-11-15

**Authors:** Saskia Kenndoff, Alexander Nieto, Julian Elias Guggenberger, Julian Taugner, Sina Mansoorian, Lukas Käsmann, Nina-Sophie Schmidt-Hegemann, Farkhad Manapov, Claus Belka, Chukwuka Eze

**Affiliations:** a Department of Radiation Oncology, University Hospital LMU Munich, Munich, Germany; b German Cancer Consortium (DKTK), Partner Site Munich; and German Cancer Research Center (DKFZ), Heidelberg, Germany; c Comprehensive Pneumology Center Munich (CPC-M), Member of the German Center for Lung Research (DZL), Munich, Germany; d Bavarian Cancer Research Center (BZKF), Munich, Germany

## Abstract

**Purpose:**

The proliferation rates of non-small cell lung cancer (NSCLC) and associated radiation resistance highlight the potential of hypofractionated radiation therapy (hypoRT). However, radiation pneumonitis and esophagitis remain dose-limiting adverse events. This study investigates dosimetric factors influencing the risk of pneumonitis and esophagitis in highly multimorbid patients undergoing moderately hypoRT.

**Methods and Materials:**

Forty-seven NSCLC patients with poor performance status treated between January 2014 and July 2021 were included. Dosimetric parameters including mean lung dose (MLD), percentage of normal (ipsi-/contralateral) lung volume (Vx) receiving ≥x Gy (x = 20, 18, 10, and 5 Gy); mean heart dose (MHD), percentage of the heart volume (HVx) receiving ≥x Gy (x = 20, 10, and 5 Gy); and mean esophageal dose (MED), percentage of esophagus volume (EVx) receiving ≥x Gy (x = 40, 30, 20, 18, 10, and 5 Gy) were analyzed retrospectively. Acute radiation pneumonitis/esophagitis events were assessed within 6/3 months posttreatment. Statistical analyses included random forests, binary logistic regression, and linear regression.

**Results:**

Among the 47 patients with compromised lung function and poor prognostic factors, 8 (17%) and 26 (55%) patients developed all-grade pneumonitis or esophagitis, while 4 (9%) and 10 (21%) patients developed CTCAE grade ≥2 pneumonitis and esophagitis, respectively. Exploratory analyses suggest that V10, V18, and MLD values are associated with an increased risk of pneumonitis. Linear regressions confirmed this for MLD values greater than 9.2 Gy (*P* = .050). Additionally, higher V5 and V10 values in the contralateral lung were associated with a greater risk of pneumonitis (*P* = .013/*P* = .032). Dmax proved to be a significant predictor of esophagitis (*P* = .020). Moreover, evidence suggests that EV5 and EV40 may portend esophagitis onset.

**Conclusions:**

This study provides insights into dosimetric factors influencing pneumonitis/esophagitis development in NSCLC patients undergoing hypoRT. While MLD and Dmax emerged as significant predictors of pneumonitis and esophagitis, the small sample size limited the depth of conclusions. Further research with larger cohorts is warranted to validate these observations, potentially optimizing treatment planning and outcomes in this challenging patient population.

## Introduction

Lung cancer remains a leading cause of mortality globally, with non-small cell lung cancer (NSCLC) comprising most diagnosed cases.^1^^,^^2^ The prognosis for NSCLC patients is typically poor, with a 5-year survival rate ranging from 10% to 20%, underscoring the urgent need for innovative treatment modalities.^3^ Platinum-based concurrent chemoradiation (cCRT) followed by maintenance therapy with the PD-L1 inhibitor durvalumab has emerged as the standard of care for inoperable stage III NSCLC. This approach has shown notable improvements in both progression-free survival (PFS) and overall survival (OS).^3^^,^^4^ However, certain patients, characterized by factors such as frailty or multimorbidity, are not viable candidates for trimodality treatment.

In rapidly proliferating tumors like NSCLC, the overall treatment time assumes critical importance. Current standard radiation therapy protocols deliver a total dose of at least 60–66 Gy (2 Gy/fraction).^5^ However, studies have demonstrated increased proliferation of NSCLC cells as early as the third to fourth week of treatment, leading to repopulation and radio-resistance.^6^^,^^7^ Hypofractionated radiation therapy (hypoRT) presents an attractive alternative by shortening the overall treatment time, potentially mitigating these effects. Moreover, tailoring the radiation schedule to the tumor's fraction sensitivity can enhance response rates and increase the biologically effective dose (BED), thereby improving local-regional control.^8^^,^^9^ Nevertheless, concerns persist regarding the safety of hypofractionated regimens, particularly in terms of severe adverse events such as pneumonitis and esophagitis.^9^, ^10^, ^11^ While extensive literature exists on dosimetric factors influencing pneumonitis development in normofractionated radiation therapy, similar analyses for hypofractionated regimens are lacking. For normofractionated chemoradiation (CRT), the percentage of normal lung volume receiving ≥20 Gy (V20), among others, is a significant predictor of grade ≥2 pneumonitis.^12^^,^^13^ The combination with immune checkpoint inhibitor (ICI)/tyrosine kinase inhibitor (TKI) therapy also showed similar results.^14^, ^15^, ^16^ The same was observed in patients receiving radiation therapy post-pneumonectomy.^17^

There is a paucity of data investigating dosimetric predictors of pneumonitis and esophagitis in patients undergoing hypofractionated regimens. To the best of our knowledge, there is a lack of studies addressing patients with compromised baseline pulmonary function. Notably, the higher BED associated with hypofractionation necessitates a reconsideration of relevant dosimetric parameters. Recent studies show the feasibility and efficacy of individualized image-guided moderately hypofractionated thoracic radiation therapy (hypo-IGRT) in patients with poor performance status and compromised pulmonary function.^18^

## Methods and Materials

### Patient characteristics

All 47 (100.0%) patients included in this study were treated at our department between January 2014 and July 2021. Inclusion criteria have been previously addressed, briefly, each patient had cytologically/histologically confirmed NSCLC and exhibited poor performance status and compromised pulmonary function, notably, forced expiratory volume in 1 second (FEV1) ≤1.0 L and/or single-breath diffusing capacity of the lung for carbon monoxide (DLCO-SB) ≤40% predicted and/or were on long-term oxygen therapy (LTOT). The total radiation dose ranged from 42.0 to 49.0 Gy, administered over 13 to 16 fractions (2.8-3.5 Gy/fraction). Detailed inclusion criteria, initial workup procedures, and the radiation therapy schedule have been previously described.^18^ Details on treatment planning were previously described in detail.^19^ Normal tissue dose-volume constraints were based on Radiation Therapy Oncology Group 0937.^20^

Our follow-up protocols were standardized according to the national guidelines.^21^ For all patients, at least a CT of the chest/upper abdomen (in some cases PET/CT) was performed every 3 months during the first 2 years. In the subsequent 2 years, the intervals were extended to every 6 months. Patients were evaluated before treatment, at least twice a week during treatment, and 4-6 weeks after hypo-IGRT to monitor acute toxicity. Acute non-hematologic toxicity was assessed using the Common Terminology Criteria for Adverse Events (versions 4.0) during treatment and up to 3 months posttreatment (up to 6 months for pneumonitis) during follow-up.

### Assessment and endpoints

We retrospectively explored the association between the development of acute radiation pneumonitis/esophagitis and dosimetric parameters. Acute events are defined as those occurring within 3 months after treatment (6 months posttreatment for pneumonitis). Statistical analyses are performed using dichotomized groups: Group A: pneumonitis/esophagitis grade ≥2 per the National Cancer Institute (NCI) Common Terminology Criteria for Adverse Events version 4.0 (CTCAE); Group B: pneumonitis/esophagitis CTCAE grades 0, 1, 2+. Collected dosimetric data included mean lung dose (MLD), percentage of normal lung volume receiving ≥20 Gy (V20), ≥18 Gy (V18), ≥10 Gy (V10), ≥5 Gy (V5); mean heart dose (MHD), percentage of the heart volume receiving ≥20 Gy/≥10 Gy/≥5 Gy (HV20/HV10/HV5); mean esophageal dose (MED); percentage of esophagus volume receiving ≥40 Gy (EV40), ≥30 Gy (EV30), ≥20 Gy (EV20), ≥18 Gy (EV18), ≥10 Gy (EV10), ≥5 Gy (EV5). In addition, dosimetric parameters of the ipsilateral and the contralateral lung (V20, V18, V10, and V5) were collected.

Furthermore, survival data from our previous analysis including median PFS and OS (defined as the time from the end of hypo-IGRT to death from any cause or to the last follow-up was updated.

### Statistical analysis

Statistical analyses were conducted using SPSS version 27 (IBM) and R version 4.2.1 in RStudio. Data processing used the dplyr package (v1.1.1), and clustered heatmaps of normalized and scaled radiation parameters were generated with ComplexHeatmap (v2.12.1), employing Euclidean distance and Ward's linkage method for row and column clustering. Clustered heatmaps are used in exploratory data analysis to visualize groups with similar characteristics. The process begins by clustering the data, typically through hierarchical clustering, and organizing it into a dendrogram. The reorganized data is then displayed as a heatmap in matrix form, where each cell is color-coded based on its value.^22^^,^^23^ This type of visualization is frequently used in genetic analyses^24^^,^^25^ but is also applicable to other types of exploratory data.

Random forests were computed using the randomForest package (v4.7-1.1), with 10,000 trees and importance and proximity parameters set to “True,” along with standard settings. Because of the low incidence of grade ≥3 adverse events, these cases were combined with grade 2 events (termed grade 2+). Variable importance measures from the random forest models were visualized using lollipop plots generated with ggplot2 (v3.4.2). Lollipop plots are a variation of bar plots, where values are depicted by circles at the end of lines extending from a baseline, typically zero. This format is particularly effective for exploratory analysis of homogeneous data, as it enhances the visibility of subtle differences in values.

Moreover, binary logistic regression was employed in group A to analyze the probability of developing pneumonitis or esophagitis, while linear regression was used in group B. These analyses evaluated the relationship between different dosimetric parameters and the occurrence of adverse events. Specifically for pneumonitis, separate analyses of the ipsilateral and contralateral lungs were conducted. Because of the small sample size and the limited number of adverse events, a multivariable analysis was not feasible. Median PFS and OS were estimated using the Kaplan–Meier method. Statistical significance was set at *P* value < .05.

## Results

A total of 47 patients were analyzed with patient characteristics presented in Table 1*.* The median age was 72.0 years (52.2-88.0 years), 27 (57.4%) were over 70.0 years and 27 (57.4%) were male. Twenty (42.6%) patients had squamous cell carcinoma, and 22 (46.8%) patients had adenocarcinoma. Most were initially diagnosed according to UICC TNM classification with stage IIIA, IIIB, and IIIC, 8 (17%), 17 (36.2%), and 12 (25.5%) patients, respectively. All patients had lymph node involvement (N+), with the majority presenting with N2 disease (51.1%), followed by N3 (29.8%) and N1 (19.1%). Thirty (63.8%) patients had an initial Charlson Comorbidity Index (CCI) of 4-6 and 17 (36.2%) over 7. All patients were current or former smokers. In 34 of 39 (87.2%) patients, DLCO-SB was ≤40% predicted, 18 of 47 (38.3%) were on LTOT, and 18 of 47 patients (38.3%) had an FEV1 ≤1 L. Before treatment, median baseline DLCO-SB was 35% predicted (range, 13.3-69.0), median FEV1 was 1.17 L (range, 0.69-2.84 L), median vital capacity was 2.34 L (range, 1.23-3.74 L)

**Table 1:** Patient characteristics, adapted from Eze et al.^18^

	Number of patients (%)
*Total*	47 (100)
*Age*	
Median	72 (52.2-88.0)
Mean (SD)	71.9 (8.6)
*Sex*	
Female	20 (42.6)
Male	27 (57.4)
*T category*	
Tx	8 (17.0)
T1	1 (2.1)
T2	8 (17.0)
T3	13 (27.7)
T4	17 (36.2)
*N category*	
N1	9 (19.1)
N2	24 (51.1)
N3	14 (29.8)
*UICC stage*	
IIB	2 (4.3)
IIIA	8 (17.0)
IIIB	17 (36.2)
IIIC	12 (25.5)
Recurrent (stage III)	8 (17.0)
*Histology*	
Squamous cell carcinoma	20 (42.6)
Non-squamous cell carcinoma	22 (46.8)
Not otherwise specified	5 (10.6)
*Location primary tumor*	
left lung	17 (36.2)
right lung	30 (63.8)
*Charlson Comorbidity Index*	
4-6	30 (63.8)
≥7	17 (36.2)
*Baseline FEV1*	
Median (range), L	1.17 (0.69-2.84)
Mean (SD), L	1.28 (0.5)
Median (range), %	47.5 (27.9-96.4)
Mean (SD), %	51 (17.1)
*Vital capacity*	
Median (range), L	2.34 (1.23-3.74)
Mean (SD), L	2.25 (0.64)
Median (range), %	67.8 (33-110)
Mean (SD), %	67.7 (14)
*Baseline DLCO-SB*	
Median, mmol/min/kPa	2.59 (1-4.7)
Mean (SD), mmol/min/kPa	2.7 (0.88)
Median predicted (range), %	35 (13.3-69)
Mean (SD), %	34.51 (10.46)
Long-term oxygen therapy	18 (38.3)
*Radiation therapy modality*	
3D-CRT	6 (12.8)
IMRT/VMAT	41 (87.2)
*PTV, cc*	
Median (range)	315.4 (83.4-1174.1)
Mean (SD)	410.8 (267.1)
*Induction systemic therapy*	
Yes	19 (40.4)
No	28 (59.6)
*Salvage systemic therapy*	
Yes	10 (21.3)
No	37 (78.7)
*Pneumonitis*	
CTCAE grade 1-5	8 (17.0)
CTCAE grade ≥2	4 (8.5)
*Esophagitis*	
CTCAE grade 1-5	26 (55.3)
CTCAE grade ≥2	10 (21.3)

*Abbreviations:* 3D-CRT = 3-dimensional-conformal radiation therapy; CTCAE = common terminology criteria for adverse events version 4.0; DLCO-SB *=* single-breath diffusing capacity of the lung for carbon monoxide; FEV1 *=* forced expiratory volume in 1 second; IMRT = intensity-modulated radiation therapy; VMAT = volumetric modulated arc therapy.

Before hypoRT, 19/47 patients (40.4%) received systemic therapy. Thereafter, all patients were reviewed by a multidisciplinary tumor board and were determined to be unsuitable for concurrent CRT. Consequently, radiation therapy was administered with palliative intent.

Six (12.8%) patients were treated with 3-dimensional-conformal radiation therapy (3D-CRT) and 41 (87.2%) with intensity-modulated radiation therapy (IMRT) or volumetric modulated arc therapy (VMAT). The median planning target volume (PTV) was 315.4 cc (range, 83.4-1174.1cc), and the mean PTV was 410.8 cc (SD, 267.1 cc). Within the observation period, pneumonitis, and esophagitis with CTCAE grade 1-5 occurred in 8 (17%) and 26 (55.3%) cases, respectively. CTCAE ≥2 was observed in 4 (9%) and 10 (21%) cases, respectively, with no CTCAE grade 5 observed.

### Survival parameters

At the cut-off date of 08/2023, 40 patients (85.1%) had disease progression and 35 had died. Median follow-up time was 35.2 months (range, 0.5-101.7 months), median PFS 10.6 months (95% CI, 8.7-12.5 months), and median OS 22.0 months (95% CI, 11.3-32.7 months). The 6-, 12-, and 18-month PFS/OS rates were 70.2%/89.4%; 40.4%/68.1%; and 23.2%/54.9%, respectively. Kaplan–Meier curves are depicted in the Figs. E1 and E2.

### Dosimetric data

Dosimetric data for all patients were collected and summarized. Notably for the lungs, mean values for various parameters were as follows: average MLD: 9.2 Gy (SD, 1.9 Gy), mean V20: 15.1% (SD, 4.8%), mean V18: 17.1% (SD, 5.0%), mean V10: 30.1% (SD, 7.1%), and mean V5: 48.4% (SD, 10.2%).

#### Heart

Average MHD: 5.4 Gy (SD, 3.2 Gy), mean HV20: 6.3% (SD, 5.4%), mean HV10: 18.4% (SD, 14.4%), and mean HV5: 31.3% (SD, 24.0%).

#### Esophagus

Average MED: 14.2 Gy (SD, 5.2 Gy), average maximum esophageal dose (Dmax): 41.0 Gy (SD, 6.9%), mean EV40: 5.9% (SD, 8.7%), mean EV30: 23.6% (SD, 15.2%), mean EV20: 33.8% (SD, 16.5%), mean EV18: 36.3% (SD, 15.5%), mean EV10: 46.6% (SD, 13.4%), and mean EV5: 54.3% (SD, 12.6%).

### Heatmaps

We visualized hierarchical clustering on the centered and scaled dosimetric data (row) of each patient (column). The dendrogram of the hierarchical clustering of patients is annotated with informative patient characteristics such as the binary occurrence of pneumonitis or esophagitis or ordinal scale of simultaneous occurrence of chronic obstructive pulmonary disease (COPD) and continuous data from lung function parameters. The annotated data, such as the baseline lung function parameters, were not used for clustering the heatmap. In the heatmap, red indicates high dosimetric values while blue saturation indicates a low value. Heatmaps allow the first assessment of the data structure. Fig. E3 in the supplements shows the hierarchical clustering of radiation esophageal dosimetric parameters. A subgroup of patients with esophagitis show high values of EV5 (%) and EV40 (%). Regarding the lung parameters (Fig. E4), the involved/ipsilateral lung expectedly showed high exposure. Some patients with pneumonitis also showed increased MLD, V10, and V18.

### Lollipop plots

To account for multicollinearity, we implemented a random forest regression to further explore the importance of dosimetric parameters on the occurrence of adverse events: pneumonitis and esophagitis. Here we visualize the random forest variable importance measures “mean decrease accuracy” and “mean decrease Gini” with lollipop plots. They are the main performance indicators for the random forest regression in relation to the binary/metric incidence of pneumonitis/esophagitis and dosimetric parameters. The mean decrease accuracy measures the impact of each individual radiation parameter variable on the accuracy of the model. The mean decrease accuracy measures how the accuracy of the regression model decreases if the parameter variable is removed. A larger mean decrease in accuracy suggests that the variable is more important. The “mean decrease Gini” measures the important predictor variables by describing their ability to increase purity in splits of decision trees. Variables with higher mean decrease Gini contribute more to accuracy and separation of classes and are therefore interpreted as more important.

A summary of the 5 most important parameters that predicted the occurrence of pneumonitis/esophagitis in group A/B is depicted in Table 2 (refer to Figs. E3-E8 for Heatmaps and Lollipop plots).

**Table 2:** Summary of the 5 most important parameters for predicting the incidence of pneumonitis/esophagitis in group A/B. The lollipop plot for each analysis is provided in the Supplements

Predicting the occurrence of pneumonitis in group A (Fig. E5)				
V10 (ccm)	PTV (ccm)	V18 (%)	Age (y)	MLD (Gy)
Predicting the occurrence of pneumonitis in group B (Fig. E6)				
V10 (ccm)	PTV (ccm)	V18 (%)	Age (y)	MLD (Gy)
Predicting the occurrence of esophagitis in group A (Fig. E7)				
EV18 (ccm)	HV5 (%)	HV5 (ccm)	EV30 (%)	EV18 (%)
Predicting the occurrence of esophagitis in group B (Fig. E8)				
EV18 (ccm)	HV5 (%)	HV5 (ccm)	EV30 (%)	EV30 (ccm)

*Abbreviations:* EV = esophagus volume; HV = heart volume; MLD = mean lung dose; PTV = planning target volume.

#### Group A

A total of 4 patients developed pneumonitis CTCAE grade ≥2. Binary logistic regression showed that the probability of developing pneumonitis CTCAE grade ≥2 was significantly increased for MLD values greater than 9.2 Gy (*P* = .050). Analyses with V20 [%], V18 [%], V10 [%] and V5 [%] showed no association. Also, the development of esophagitis could not be related to significantly higher risk by increasing MED, Dmax, EV40, EV30, EV20, EV18, EV10, or EV5 (*P* = .273; *P* = .095; *P* = .064; *P* = .101; *P* = .419; *P* = .584; *P* = .741; *P* = .391). All results are summarized in Table 3.

**Table 3:** Group A—*P* values of binary logistic regression from different dosimetric parameters predicting the development of pneumonitis/esophagitis grade ≥2. The significant results are marked in bold, with a *P* value <.05 demonstrating significance

Pneumonitis CTCAE grade ≥2		Esophagitis CTCAE grade ≥2	
MLD [Gy]	**0.050**	MED [Gy]	0.273
V20 [%]	0.066	D_max_ Esoph. [Gy]	0.095
V18 [%]	0.079	EV40 [%]	0.064
V10 [%]	0.341	EV30 [%]	0.101
V5 [%]	0.204	EV20 [%]	0.419
		EV18 [%]	0.584
		EV10 [%]	0.741
		EV5 [%]	0.391

Abbreviations: CTCAE = common terminology criteria for adverse events version 4.0; EV = esophagus volume; MED = mean esophageal dose; MLD = mean lung dose; V = lung volume.

Increasing values of V5 and V10 in the primarily uninvolved lung were significant for the development of pneumonitis with *P* = .013 and *P* = .032. The remaining parameters had no significance (MLD: *P* = .795; V20: *P* = .939; V18 *P* = .923). Also, binary logistic regression with values of the ipsilateral lung showed no significance (*P* = .343; *P* = .534; *P* = .490; *P* = .759; *P* = .351), see Table 4.

**Table 4:** Group A—*P* values of binary logistic regression from different dosimetric parameters predicting the development of pneumonitis grade ≥2 in the primarily involved or uninvolved lung. Significance is demonstrated by *P* value <.05 and is marked in bold

Pneumonitis CTCAE grade ≥2			
Ipsilateral/Involved Lung		Contralateral/Uninvolved lung	
MLD [Gy]	0.343	MLD [Gy]	0.795
V20 [%]	0.534	V20 [%]	0.939
V18 [%]	0.490	V18 [%]	0.923
V10 [%]	0.759	V10 [%]	**0.013**
V5 [%]	0.351	V5 [%]	**0.032**

*Abbreviations:* CTCAE = common terminology criteria for adverse events version 4.0; MLD = mean lung dose; V = lung volume.

#### Group B

Linear regression was performed and showed significance for the development of esophagitis CTCAE grade 0-5 with increasing Dmax (*P* = .020). The remaining values had no significant association (MED: *P* = .216; EV40: *P* = .328; EV30: *P* = .075; EV20: *P* = .141; EV18: *P* = .231; EV10: *P* = .935; EV5: *P* = .213). Regarding the risk of pneumonitis CTCAE 0-5, increasing MLD [Gy], V20 [%], V18 [%], V10 [%], and V5 [%] showed no significant association. Results are shown in Table 5.

**Table 5:** Group B—*P* values of linear regression representing the influence of different dosimetric parameters on the development of pneumonitis/esophagitis CTCAE grade 0-5. The significant results are marked in bold, with a *P* value < .05 demonstrating significance

Pneumonitis CTCAE grade 0-5		Esophagitis CTCAE grade 0-5	
MLD [Gy]	0.299	MED [Gy]	0.216
V20 [%]	0.357	D_max_ Esoph. [Gy]	**0.020**
V18 [%]	0.458	EV40 [%]	0.328
V10 [%]	0.940	EV30 [%]	0.075
V5 [%]	0.623	EV20 [%]	0.141
		EV18 [%]	0.231
		EV10 [%]	0.935
		EV5 [%]	0.213

Abbreviations: CTCAE = common terminology criteria for adverse events version 4.0; EV = esophagus volume; MED = mean esophageal dose; MLD = mean lung dose; V = lung volume.

Linear regressions with the primary involved lung were not significant in group B. Analyses of the uninvolved lung showed significance for V5 and V10 with *P* = .003 and *P* = .018, respectively. The remaining parameters were not significant (MLD: *P* = .618; V20: *P* = .824; V18: *P* = .849) (summarized in Table 6).

**Table 6:** Group B—*P* values of linear regression with different dosimetric parameters predicting the development of pneumonitis grade 0-5 in the primarily involved or uninvolved lung. Significance is demonstrated by *P* value < .05 and marked bold

Pneumonitis CTCAE grade 0-5			
Involved lung		Uninvolved lung	
MLD [Gy]	0.28	MLD [Gy]	0.618
V20 [%]	0.779	V20 [%]	0.824
V18 [%]	0.752	V18 [%]	0.849
V10 [%]	0.884	V10 [%]	**0.003**
V5 [%]	0.569	V5 [%]	**0.018**

Abbreviations: CTCAE = common terminology criteria for adverse events version 4.0; MLD = mean lung dose; V = lung volume.

## Discussion

Hypofractionated radiation therapy has gained increasing attention as an alternative approach to escalate the BED while reducing treatment time and costs, particularly for patients with NSCLC and poor prognostic factors.^9^^,^^26^^,^^27^ Our research group recently demonstrated promising outcomes for highly multimorbid patients treated with hypofractionated radiation therapy (RT), further supporting the safety and feasibility of this treatment regimen.^19^^,^^28^

The updated survival outcomes, including 6-, 12-, and 18-month PFS and OS rates were 70.2%/89.4%, 40.4%/68.1%, and 23.2%/54.9%, respectively, underscoring the safety and feasibility of this hypofractionated treatment regimen.^18^, ^19^ These results are consistent with the growing body of evidence supporting the efficacy of hypoRT in NSCLC patients with poor prognostic factors. In a phase 3 randomized trial conducted by Iyengar et al,^29^ patients with stage II/III NSCLC and poor performance status were allocated to receive either normofractionated or hypofractionated (60 Gy/4 fx). The study reported a 1-year OS rate of 37.7% in the hypofractionated arm.^29^ Notably, 5 out of 50 patients (10%) in the hypofractionation arm died during treatment.^29^ Consequently, after carefully weighing the risks, we decided to proceed with a risk-adapted dose concept for our patients. Our results demonstrate improved outcomes albeit in a retrospective cohort, further supporting the efficacy of hypoRT in this patient population. Despite its potential benefits, dose-limiting toxicities such as radiation pneumonitis and esophagitis remain significant concerns.^11^^,^^30^

Technological advancements in radiation techniques aim to optimize dose distribution and spare surrounding healthy tissues.^31^, ^32^, ^33^ While parameters like MLD and percentage of normal lung volume receiving specific doses (Vx) are commonly discussed in normofractionated RT/CRT series,^16^^,^^34^, ^35^, ^36^, ^37^, ^38^, ^39^, ^40^ our exploratory analysis aimed to identify dosimetric parameters associated with pneumonitis/esophagitis development in this distinct patient cohort with baseline compromised pulmonary function. Our exploratory analysis revealed associations between higher V10, V18, and MLD values approaching significance and increased risk of pneumonitis in our multimorbid patient cohort. Notably, our findings suggest that stringent reduction of MLD and V18 may mitigate the risk of pneumonitis, aligning with studies emphasizing the importance of limiting radiation parameters to minimize toxicity. McFarlane et al^38^ reported lung V5 metric as a significant predictor of G2+ pneumonitis and MLD and V20 for G3+ pneumonitis in a statewide quality consortium study including data of patients treated with conventionally fractionated radiation therapy from 27 academic and community clinics. However, the complex interplay between various dosimetric and clinical factors underscores the need for comprehensive analyses and predictive models to optimize treatment planning and outcomes.^37^^,^^41^ The use of models to calculate the probabilities of complications in normal tissue (NTCP) has emerged as a valuable tool in radiation oncology. Niezink et al^42^ demonstrated that combining dosimetric parameters such as MLD with patient-specific factors such as age and smoking status improved the predictive power for radiation pneumonitis in patients undergoing IMRT or VMAT. This approach highlights the importance of integrating both dosimetric and clinical factors to better predict and mitigate treatment-related toxicities. By refining existing NTCP models and incorporating patient-specific characteristics, clinicians can tailor treatment plans to minimize the risk of complications, ultimately optimizing patient outcomes.^42^ Moreover, the functional inhomogeneity of the lung contributes to the likelihood of pulmonary injury. Certain areas are identified as more relevant than others concerning ventilation/perfusion, causing them to be potentially more affected by high doses than other areas.^43^^,^^44^ This has been emphasized by numerous studies demonstrating that lung functional avoidance RT is linked to reduced pulmonary injuries.^45^^,^^46^

In our data set, our analysis reveals that V10(ccm), PTV, V18, age, and MLD emerge as the most significant predictors of pneumonitis. These variables are integral in evaluating the probability of developing pneumonitis following hypoRT. Interestingly, our findings align with a recent study suggesting strategies to mitigate the risk of grade ≥2 radiation pneumonitis. Specifically, maintaining lung V20 <17.7%, MLD <10.6 Gy, and V5 <41.3% when administering 4 Gy per fraction to 60-72 Gy is recommended.^47^ Our data set also suggests that higher values of V5 and V10, particularly in the contralateral lung, are associated with an increased likelihood of developing pneumonitis, potentially serving as surrogates for whole-lung exposure. Conversely, we did not observe a correlation with the dose to the ipsilateral lung, likely because of the small sample size of our cohort. Existing literature supports the association between dose to the whole lung and pneumonitis onset.^48^^,^^49^ In summary, we propose that limiting the MLD to <9.2 Gy in hypoRT treatments effectively reduces the risk of pneumonitis. When compared to the NRG-LU004 study^50^ and other hypofractionation studies including treatments with 15 fractions as outlined in Table E1, our constraints are notably more conservative (average MLD <10 Gy).^20^^,^^29^^,^^47^^,^^50^, ^51^, ^52^, ^53^, ^54^, ^55^, ^56^ This may stem from our smaller sample size and low incidence of adverse events. Additionally, the high levels of multimorbidity and severely impaired lung function of our patients influenced our decision to adopt more cautious constraints. Consequently, the recommendations we provide could likely be broadened and should primarily serve as guidance for hypofractionation concepts in multimorbid patients with compromised pulmonary function. Furthermore, while Sasse et al^47^ suggested similar more conservative MLD constraints, this highlights the need for further validation in the hypofractionation setting.

Additionally, both V10 and V18 demonstrate predictive potential. However, caution is warranted as our analysis, given the small sample size, can only be considered exploratory. Validation in a larger cohort is necessary, although challenging given the unique characteristics of this patient population. Furthermore, the integration of dosimetric parameters with clinical factors is essential for developing models with enhanced predictive power.

When considering treatment-limiting side effects in thoracic radiation therapy, the occurrence of esophagitis emerges as particularly significant. Previous research, exemplified by Belderbos et al,^57^ has underscored the impact of metrics like EV35 as a dosimetric predictor of esophageal toxicity in patients receiving hypofractionated (chemo-)radiation (2.25-2.75 Gy/fx up to 49.5-94.5 Gy). In the PET-Boost trial, conducted by the same group, patients were treated with an isotoxic integrated boost of ≥72 Gy delivered in 24 fractions, with or without chemotherapy and adherence to strict dose limits. However, the personalized dose-escalation approach resulted in higher rates of acute and late toxicity compared to conventional chemoradiation (CRT), with several patients experiencing fatal pulmonary hemorrhages and esophageal fistulae.^58^

In a further analysis by the same statewide consortium, as mentioned previously for pneumonitis, D2cc rather than MED emerged as one of the most predictive dose metric for severe esophagitis. Our exploratory analysis indicated potential relevance for EV18 and EV30 in our cohort. However, linear regressions did not corroborate these results, with only Dmax emerging as a significant factor.

Based on these findings, further analysis on larger cohorts of multimorbid patients with limited general health status undergoing hypoRT is essential to identify relevant predictors accurately. One potential strategy could involve incorporating artificial intelligence. A recently published secondary analysis of the Radiation Therapy Oncology Group (RTOG) 0617 trial demonstrated that machine-learning approaches validated known dosimetric thresholds and outperformed logistic regression in predicting toxicity. Moreover, using explainable artificial intelligence could help identify clinically useful dosimetric thresholds, which could then be externally validated.^59^

We acknowledge the limitations of our study, including potential selection bias resulting from recruitment from a single institution and a limited number of patients. The retrospective study design also presents limitations. Additionally, the analyses conducted are exploratory based on the low incidence rates of grade ≥2 pneumonitis/esophagitis (8.5%/21.3%). Despite these limitations, our findings are encouraging and suggest a clinical pathway for the management of multimorbid patients who are not amenable to traditional treatment protocols. Further research incorporating larger patient populations is warranted to validate and expand on our observations.

## Conclusions

In conclusion, our study of highly multimorbid patients sheds light on dosimetric factors that may contribute to pneumonitis and esophagitis development in hypofractionated treatment approaches. While our findings suggest the relevance of MLD and V18 in predicting pneumonitis occurrence, the small sample size limits the depth of our conclusions to general trends. Further research involving larger cohorts is essential to validate and refine these observations, ultimately enhancing our understanding of treatment outcomes in this patient population.

## Disclosures

The Department of Radiation Oncology of the LMU University Hospital, LMU Munich has research agreements with ViewRay Inc Elekta, Brainlab, and C-RAD outside the submitted work. Saskia Kenndoff, Alexander Nieto, Julian Taugner, Sina Mansoorian, Nina-Sophie Schmidt-Hegemann, and Julian Elias Guggenberger, report no conflicts of interest. Lukas Käsmann reports a research grant from AstraZeneca (unrestricted research grant [NCT05027165]), AMGEN, Art Tempi (MasterClass LucaNext 2023/2024) outside the submitted work, and honoraria from the German Cancer Society, AstraZeneca, and Art Tempi outside the submitted work. Lukas Käsmann received support for attending meetings and/or travel from AstraZeneca and ELCC outside the submitted work. Farkhad Manapov reports a research grant from AstraZeneca and honoraria from AstraZeneca, Novartis, Roche, Lilly, Elekta, and Brainlab outside the submitted work, receiving support for attending meetings or travel from AstraZeneca, Elekta, and Brainlab outside the submitted work. Farkhad Manapov serves on the advisory board of AstraZeneca, Novartis. Claus Belka reports receiving grants or contracts from ViewRay, Brainlab, and Elekta; payment or honoraria from Bristol-Myers Squibb, Roche, Merck, AstraZeneca, Elekta, and ViewRay; receiving support for attending meetings or travel from Bristol-Myers Squibb, Roche, Merck, AstraZeneca, Elekta, and ViewRay; and having a leadership or fiduciary role with ESTRO, all outside the submitted work. Chukwuka Eze received funding in the form of a research grant from the German Cancer Aid; reports consulting fees from Novartis outside the submitted work and Associate Editor role with Heliyon.

## Appendix Supplementary materials

Supplements_291024 Image, application 1

## Footnotes

Supplementary material associated with this article can be found in the online version at doi:10.1016/j.adro.2024.101682.
