# Supplementary material for: Dosimetric Predictors of Acute Radiation Pneumonitis and Esophagitis in Hypofractionated Thoracic Irradiation of Non-Small Cell Lung Cancer Patients With Poor Prognostic Factors
Source: Adv Radiat Oncol. 2024 Nov 15;10(2):101682. doi: 10.1016/j.adro.2024.101682 (PMC11786736; doi:10.1016/j.adro.2024.101682)
Supplement: Supplements_291024 [file mmc1.docx]

**Supplements**


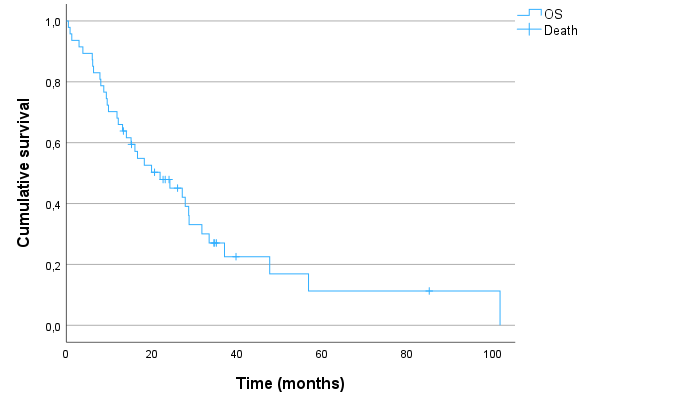


**Figure 1:** Overall survival (OS)


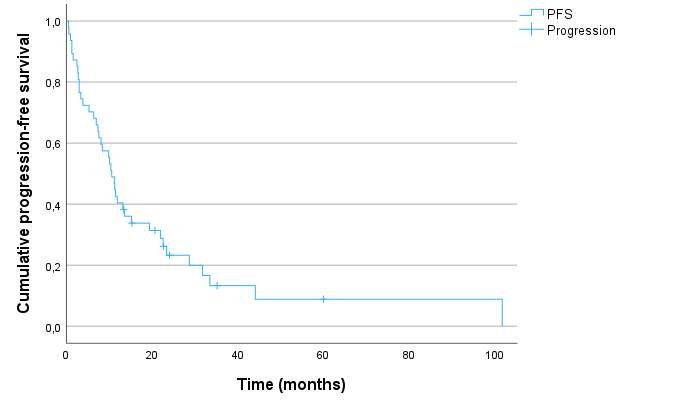


**Figure 2:** Progression-free survival (PFS)


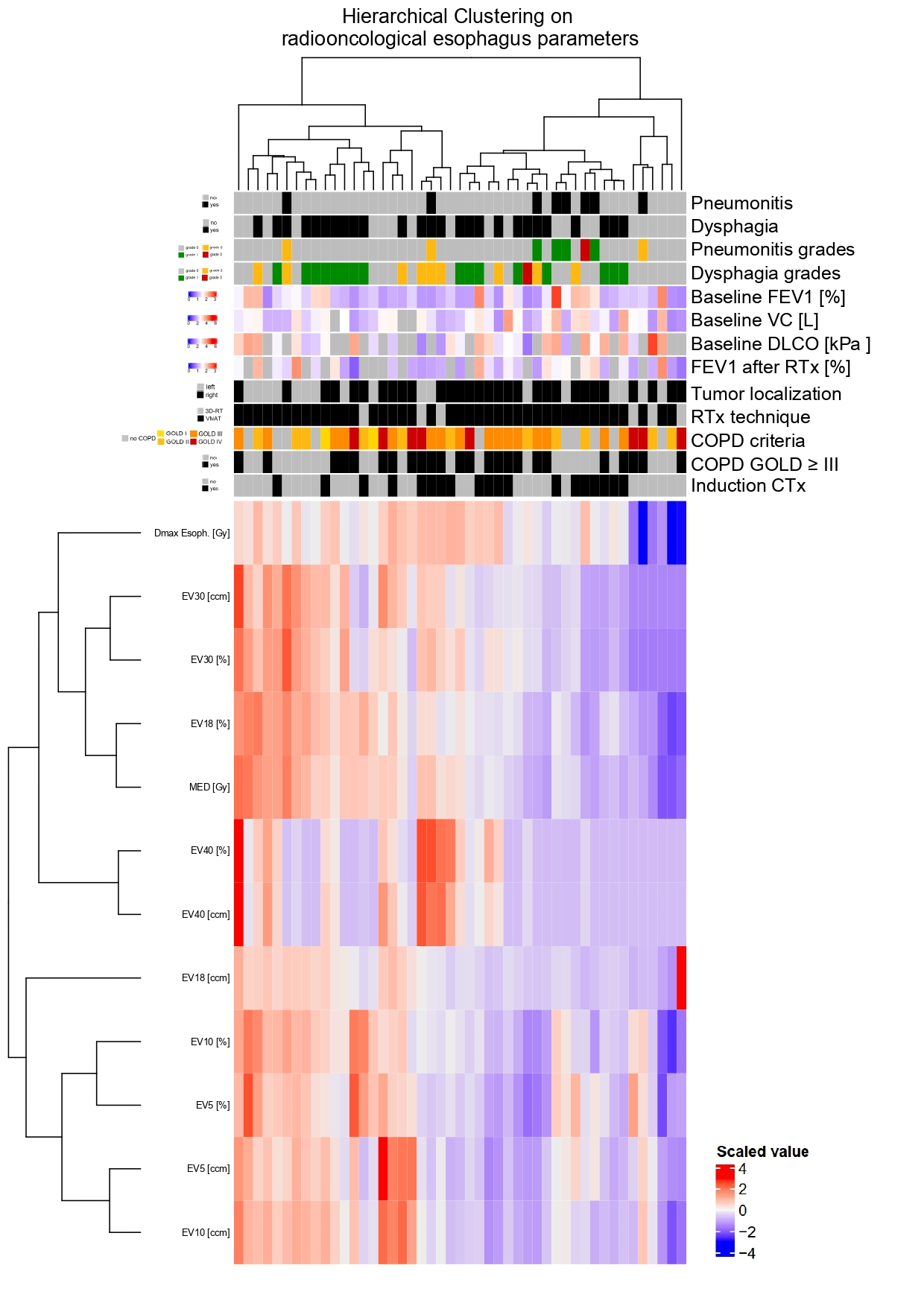


**Figure 3:** Heatmap with hierarchical clustering of dosimetric parameters of the oesophagus. Each column represents a patient, and each row represents a specific characteristic. The dendrogram at the top shows the clustered patients based on patient characteristics, while the dendrogram at the bottom shows the clustering on dosimetric oesophageal parameters. [RTx= radiotherapy, COPD= chronic obstructive pulmonary disease, CTx= chemotherapy, FEV1= forced expiratory volume in 1 second, VC= vital capacity, DLCO= diffusing capacity of the lungs for carbon monoxide, MED= mean oesophageal dose, EVx= percentage of the oesophagus volume receiving 40 Gy / 30 Gy / 18 Gy / 10 Gy / 5 Gy or more].


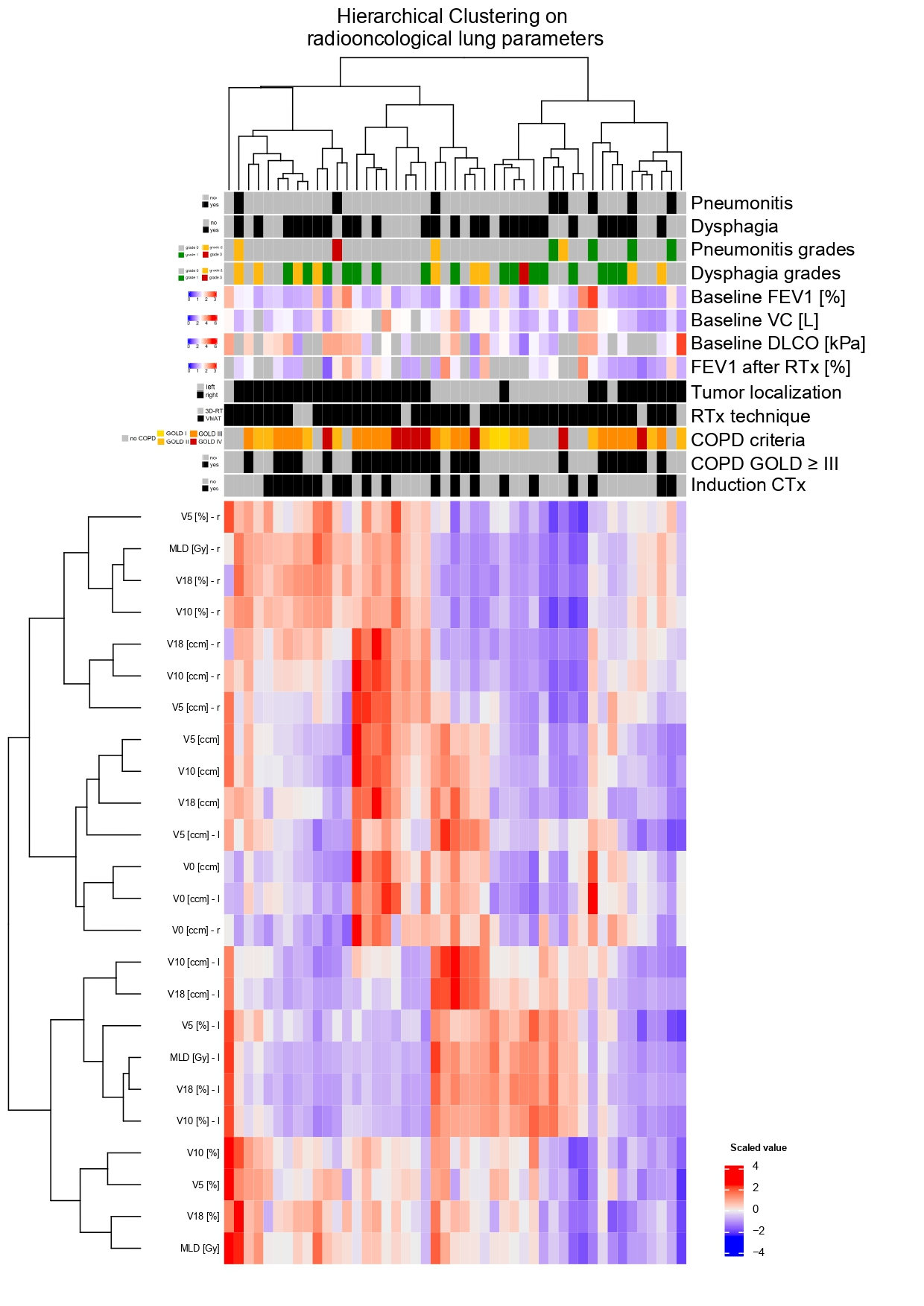


**Figure 4:** Heatmap with hierarchical clustering of dosimetric parameters of the lung. Each column represents a patient, and each row represents a specific characteristic. The dendrogram at the top shows the clustered patients based on patient characteristics, while the dendrogram at the bottom shows the clustering on dosimetric oesophageal parameters. [RTx= radiotherapy, COPD= chronic obstructive pulmonary disease, CTx= chemotherapy, FEV1= forced expiratory volume in 1 second, VC= vital capacity, DLCO= diffusing capacity of the lungs for carbon monoxide, l= left, r= right, MLD= mean lung dose, Vx= percentage of the lung volume receiving 18 Gy / 10 Gy / 5 Gy or more].


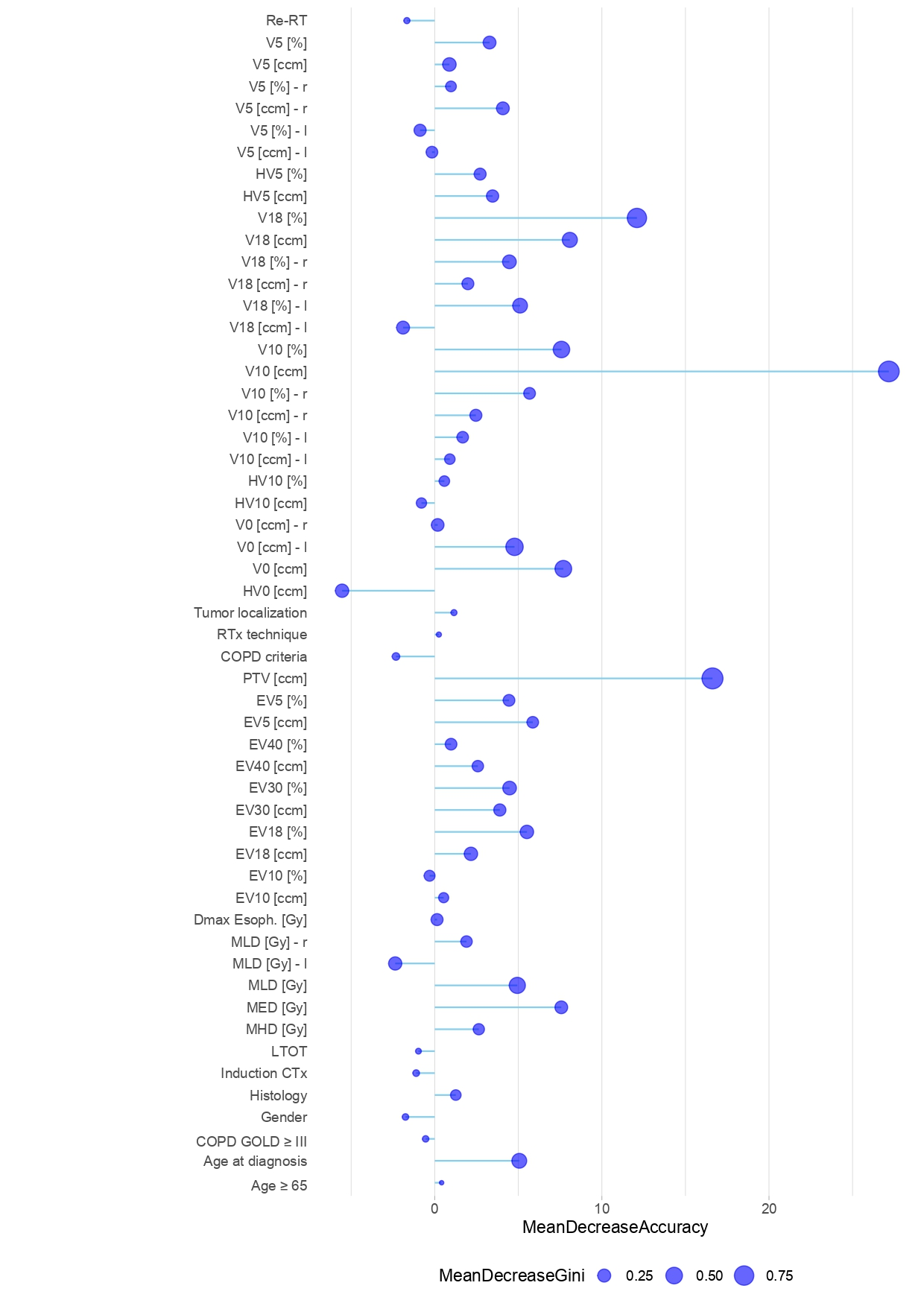


**Figure 5:** Lollipop-plot showing the main performance indicators for the random forest regression in relation to the binary incidence of pneumonitis and dosimetric parameters in group A. The higher the mean decrease gini, the higher the accuracy of class separation and therefore interpreted as more important. [Re-RT= reirradiation, RTx= radiotherapy, COPD= chronic obstructive pulmonary disease, LTOT= long term oxygen therapy, PTV= planning target volume, MLD= mean lung dose, MED= mean oesophageal dose, MHD= mean heart dose, Vx= percentage of the lung volume receiving 18 Gy / 10 Gy / 5 Gy / 0Gy or more, HVx= percentage of the heart volume receiving 10 Gy / 5 Gy / 0 Gy or more, EVx= percentage of the oesophagus volume receiving 40 Gy / 30 Gy / 18 Gy / 10 Gy / 5 Gy or more].


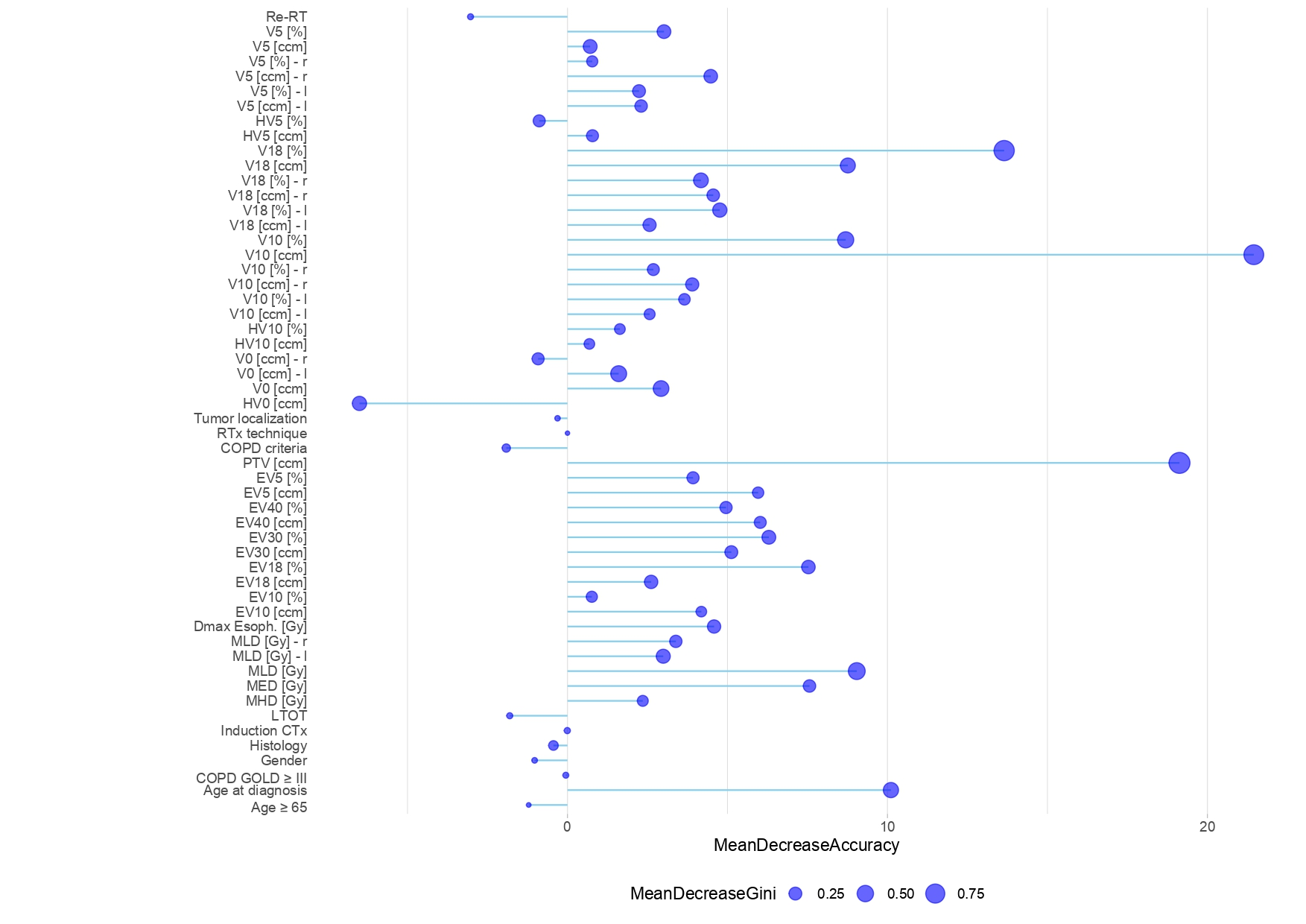


**Figure 6**: Lolipop-plot showing the main performance indicators for the random forest regression in relation to the occurrence of pneumonitis incorporating pneumonitis grade information (grade 0, grade 1, grade 2+) and dosimetric parameters in group A. The higher the mean decrease gini, the higher the accuracy of class separation and therefore interpreted as more important. [Re-RT= reirradiation, RTx= radiotherapy, COPD= chronic obstructive pulmonary disease, LTOT= long term oxygen therapy, PTV= planning target volume, MLD= mean lung dose, MED= mean oesophageal dose, MHD= mean heart dose, Vx= percentage of the lung volume receiving 18 Gy / 10 Gy / 5 Gy / 0 Gy or more, HVx= percentage of the heart volume receiving 10 Gy / 5 Gy / 0 Gy or more, EVx= percentage of the oesophagus volume receiving 40 Gy / 30 Gy / 18 Gy / 10 Gy / 5 Gy or more].


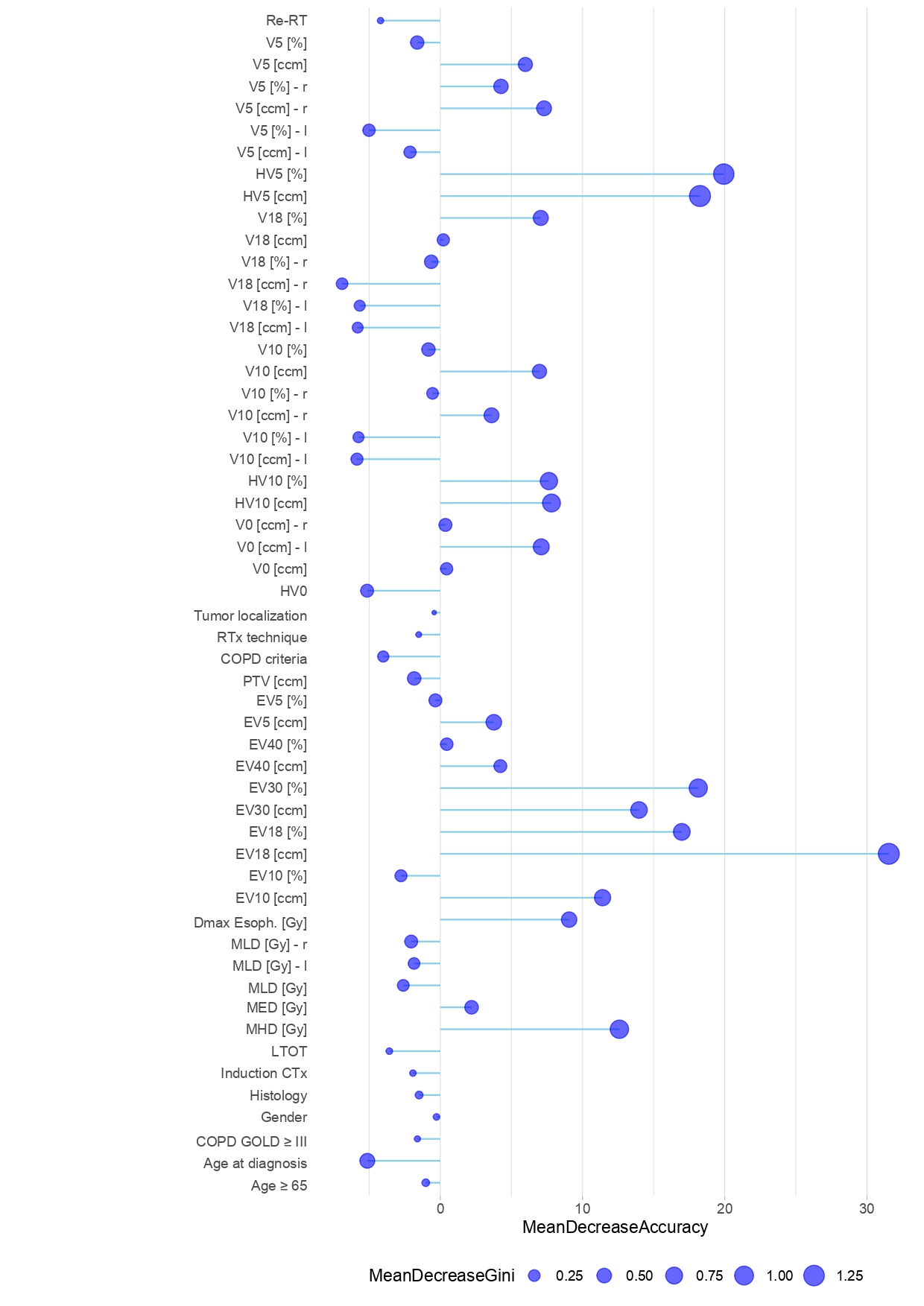


**Figure 7:** Lolipop-plot showing the main performance indicators for the random forest regression in relation to the binary incidence of dysphagia/oesophagitis and dosimetric parameters in group A. The higher the mean decrease gini, the higher the accuracy of class separation and therefore interpreted as more important. [Re-RT= reirradiation, RTx= radiotherapy, COPD= chronic obstructive pulmonary disease, LTOT= long term oxygen therapy, PTV= planning target volume, MLD= mean lung dose, MED= mean osophageal dose, MHD= mean heart dose, Vx= percentage of the lung volume receiving 18 Gy / 10 Gy / 5 Gy / 0 Gy or more, HVx= percentage of the heart volume receiving 10 Gy / 5 Gy / 0 Gy or more, EVx= percentage of the oesophagus volume receiving 40 Gy / 30 Gy / 18 Gy / 10 Gy / 5 Gy or more].


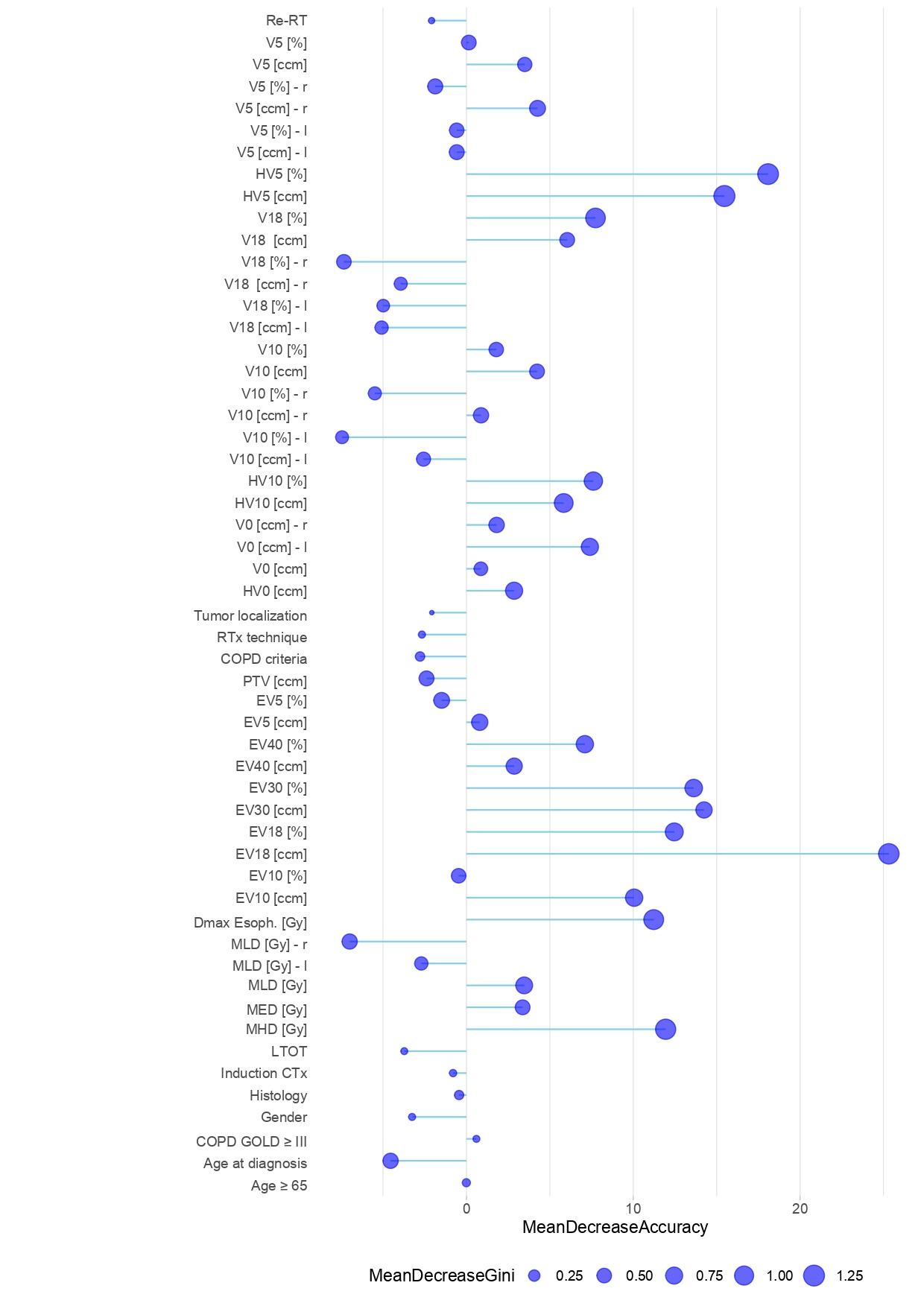


**Figure 8:** Lolipop-plot showing the main performance indicators for the random forest regression in relation to the occurrence of dysphagia/oesophagitis incorporating dysphagia/oesophagitis grade information (grade 0, grade 1, grade 2+) and dosimetric parameters in group A. The higher the mean decrease gini, the higher the accuracy of class separation and therefore interpreted as more important. [Re-RT= reirradiation, RTx= radiotherapy, COPD= chronic obstructive pulmonary disease, LTOT= long term oxygen therapy, PTV= planning target volume, MLD= mean lung dose, MED= mean oesophageal dose, MHD= mean heart dose, Vx= percentage of the lung volume receiving 18 Gy / 10 Gy / 5 Gy / 0 Gy or more, HVx= percentage of the heart volume receiving 10 Gy / 5 Gy / 0 Gy or more, EVx= percentage of the oesophagus volume receiving 40 Gy / 30 Gy / 18 Gy / 10 Gy / 5 Gy or more].

| **Ref.** | **TD (Gy)** | **D/fx** | **Lung V5 (%)** | **Lung V16.5 (%)** | **Mean Lung Dose (Gy)** | **Esophagus Dmax (Gy)** | **Esophagus Mean (Gy)** | **Heart Dmax (Gy)** | **Heart Mean (Gy)** | **Esophagitis ≤ Grade 2 (%)** | **Esophagitis > Grade 2 (%)** | **Pneumonitis ≤ Grade 2 (%)** | **Pneumonitis > Grade 2 (%)** | **Comments** |
| --- | --- | --- | --- | --- | --- | --- | --- | --- | --- | --- | --- | --- | --- | --- |
| (Sasse et al. 2024) | 60-72 | 4 | </=41.3* | </=20.2* | </=10.6* | - | - | - | - | - | - | - | 16.8** | *Maintaining grade </=20%  ** >/= grade 2 |
| (Giuliani et al. 2024) | 60 | 4 | - | - | </= 14 | </=50.5 | - | </=66 | - | 13.3 | 0 | 10 | 3 |  |
| (Swaminath et al. 2024) | 60 | 4 | - | - | </= 18 | </=48 | - | </=66 | - | Toxic effects ≤ grade 2 (32%); > grade 2 (1%) |  |  |  |  |
| (Puckett et al. 2023)* | 60 | 4 | </=65 | <37 | </= 16.5 | </=58.9 | </=25 | </=60 | >16.5 | - | - | - | - | *Recommendation by VA and ASTRO based on Lin et al. and expert consensus |
| (Timmerman 2022) | 60 | 4 | - | <37 | </= 16.5 | </=48 | - | </=48.9 | - | - | - | - | - | - |
| (Lin et al. 2021) | 60 | 4 | </=65 | <37 | </= 16.5 | </=58.9** | </=31 | </=49.8** | >16.5 | - | - | - | - | **D0.03cc(Gy) |
| (Iyengar et al. 2021) | 60 | 4 | - | <37* | - | </=55.3^§^ | - | </=48.9^§^ | - | 38 | 2 | 8 | 2 | *V18  ^§^D0.035cc (Gy) |
| (Gore et al. 2017) | 45 | 3 | - | </=30* | </= 20 | 105% of described dose | - | - | - | - | - | - | - | *V20 |
| (Westover et al. 2015) | 60 | 4 | - | <37* | </= 18 | </=55* | - | </=49** | - | 94.7 | 5.3 | - | - | *V18  **D0.035cc (Gy) |
| (Strøm et al. 2013) | 45 | 3 | - | - | - | - | - | - | - | 56.7 | 30 | - | - | - |

**Table 1**: Summary of various studies with hypofractionated irradiation or hypofractionated irradiated cohorts and their dose constraints. TD = Total dose; Vx = percentage of the volume receiving xGy or more; Dmax = maximum dose; VA =Veterans Affairs; ASTRO = American Society for Radiation Oncology, DVH = dose-volume histogram
